# Long non-coding RNA HOTAIR promotes osteoarthritis progression via miR-17-5p/FUT2/β-catenin axis

**DOI:** 10.1038/s41419-018-0746-z

**Published:** 2018-06-15

**Authors:** Jialei Hu, Zi Wang, Yujia Shan, Yue Pan, Jia Ma, Li Jia

**Affiliations:** 1 College of Laboratory Medicine, Dalian Medical University, Dalian, Liaoning Province 116044 China; 2 Department of Sports Medicine, Dalian Municipal Central Hospital, Dalian, Liaoning Province 116033 China

## Abstract

Osteoarthritis (OA) is a chronic joint disease and hard to cure at present. Accumulating evidence suggests long noncoding RNA-HOTAIR (lncRNA-HOTAIR) plays important role in OA progression. However, the underlying molecular mechanism of HOTAIR in OA progression has not been well elucidated. In the present study, we identified that HOTAIR level was upregulated in OA cartilage tissues. High expression of HOTAIR was correlated with modified Mankin scale, extracellular matrix (ECM) degradation and chondrocytes apoptosis. The expression of miR-17-5p was down-regulated, while alpha-1, 2 fucosyltransferase 2 (FUT2) was increased in OA progression. Luciferase reporter and RNA immunoprecipitation (RIP) assays indicated that HOTAIR could directly bind to miR-17-5p and indirectly upregulate FUT2 level. Functional investigation revealed HOTAIR and FUT2 aggravated ECM degradation and chondrocytes apoptosis, and this effect could be reversed by miR-17-5p. Altered FUT2 modulated the activity of wnt/β-catenin pathway and HOTAIR/miR-17-5p also mediated wnt/β-catenin pathway through FUT2. Collectively, our findings indicated that HOTAIR/miR-17-5p/FUT2 axis contributed to OA progression via wnt/β-catenin pathway, which might provide novel insights into the function of lncRNA-driven in OA.

## Introduction

Osteoarthritis (OA) is characterized by the destruction of articular cartilage, which is mainly due to the imbalance of extracellular matrix components such as type ⍺ collagen and proteoglycan. The degradation of extracellular matrix is mainly owning to the activation of matrix metalloproteinase (MMPs) and a disintegrin and metalloproteinase domain with thrombospondin motifs family (ADAMTSs) proteins^1,2^. Patients with OA suffer from many problems, such as joint pain, temporary stiffness, joint crepitus, periarticular tenderness, swelling and limited joint function, which may lead to obstacles in activities of daily living^3,4^. Although the treatment is constantly improving, OA is still hard to cure. Therefore, it is necessary to explore the underlying molecular mechanism of OA.

Long non-coding RNAs (lncRNAs) are a series of over 200 nucleotides noncoding endogenous RNAs, which have been confirmed to play important roles in the development of inflammation-related diseases^5^. Recently, more and more evidence demonstrated that lncRNAs were involved in cell biological processes including cell proliferation and apoptosis, cellular differentiation, tumorigenesis, and metastasis^6,7^. MicroRNAs (miRNAs), a series of small noncoding RNAs with 18–22 nt in length, also have been reported in inflammation-related diseases^8^. Recent literature has documented that lncRNAs enriched in the cytoplasm typically participated in post-transcriptional regulation by interacting with miRNAs or mRNAs^9–11^. HOTAIR promoted cell proliferation, cell cycle progression, invasion and malignancy by suppressing miR-148b-3p in glioma cells^12^. Liu et al.^13^ reported that HOTAIR functioned as a competing endogenous RNA (ceRNA) of miR-331-3p to regulate HER2 expression in gastric cancer. In regard to OA, several studies demonstrated the expression of HOTAIR was aberrant upregulated during OA progression. However, the functional role of HOTAIR and underlying mechanism in OA development remained unclear.

Glycosylation is a common form of protein modification, which regulates the function of protein and plays a pivotal role in many biological processes^14^. The fucosyltransferase (FUT) family is a group of fucosylated synthetase, which transfers the catalyzed fucose from GDP fucose to oligosaccharides, sugar chains of glycoproteins or glycolipids on the substrate^15,16^. Our previous study reported FUT1, FUT2, and FUT4 were aberrant upregulated in OA cartilage tissues^17^. Many studies reported that FUT2 functioned in the pathogeny and the mechanism of primary sclerosing cholangitis, ulcerative colitics, acute gastroenteritics and other diseases^18^. However, the role of FUT2 in OA was still not documented. The wnt/β-catenin pathway was typically quiescent in many adult organs and was activated during injury^19^. Its role in tissue repair and regeneration was complex and not fully understood, although more and more data indicated that its activation enhanced fibrotic repair^20^. It was well known that the activity of wnt/β-catenin pathway aggravated OA progression. Understanding wnt/β-catenin pathway would lead to more effective diagnosis and treatment.

In this study, we identified that overexpressed HOTAIR was a characteristic molecular change in OA pathogenesis and explored the biological role of HOTAIR on the phenotype of chondrocytes. Furthermore, mechanistic analysis revealed that HOTAIR positively regulated FUT2 through sponging miR-17-5p. The HOTAIR/miR-17-5p could regulate the activity of wnt/β-catenin pathway via FUT2 in OA progression. Our findings suggested HOTAIR/ miR-17-5p/FUT2/β-catenin axis might serve as a predictive biomarker in OA treatment.

## Materials and methods

### Clinical samples

OA cartilage tissues were collected from OA patients who underwent total knee replacement surgery (*n* = 80, age 67.3 ± 8.5 years). Normal cartilage tissues were obtained from patients who were underwent the amputation without OA or rheumatoid arthritis history (*n* = 12, age 42.3 ± 6.2 years). The diagnosed of these people were according to the American College of Rheumatology criteria^21^. After surgery completed, tissue samples were frozen in liquid nitrogen immediately until usage. This study was approved by the Research Ethics Committee of Dalian Central Hospital and Dalian Medical University. All participants read and signed the informed consents.

### Cell culture

Chondrocytes were extracted from OA cartilage tissues, as previously described^22^. In brief, cartilage specimen was dissected into small sections and subjected to sequential digestion with 0.1% trypsin (Invitrogen, Carlsbad, CA, USA) for 30 min and then with 0.2% collagenase Type II (Millipore Corp., Billerica, MA, USA) in Dulbecco’s modified Eagle’s medium (DMEM) (Gibco) for 10 h at 37 °C. Undigested tissue was separated from cells using a 40 mm filter. Chondrocytes were isolated after centrifugation. Cells were maintained in DMEM (Gibco) containing 10% fetal bovine serum in incubator at 37 °C with 5% CO_2_. All the experiments were done within the second and third passage cultured chondrocytes.

### RNA extraction and quantitative real-time PCR

Total RNAs from human cartilage tissues and cultured chondrocytes were isolated using the TRIzol reagent and reversed transcribed. Quantitative real-time PCR (qRT-PCR) was done by using SYBR GreenMix (Takara) on the Biosystems 7300 Real-Time PCR system (ABI, Foster City, CA, USA). Relative gene expression was calculated using the ΔΔCt method. Relative lncRNA and miRNA expression was normalized to the U6 expression and the expression of mRNA level was normalized to GAPDH.

### Cell transfection and IL-1β stimulation

For overexpression of HOTAIR and FUT2, HOTAIR or FUT2 cDNA was cloned into the multiple cloning site of the pcDNA3.1 vector (Invitrogen, Carlsbad, CA, USA). MiR-17-5p mimic, negative control oligonucleotides (miR-NC), miR-17-5p inhibitor, negative control oligonucleotide (NC inhibitor), small interfering RNA of HOTAIR or FUT2 (siHOTAIR, siFUT2), scramble siRNA of HOTAIR or FUT2 (siSCR) were purchased from RiboBio (Guangzhou, China). The chondrocytes were seeded into 6-well plates and transfection was performed by using Lipofectamine 3000 (Invitrogen, Carlsbad, CA, USA). After 48 h of transfection, chondrocytes were stimulated with IL-1β (10 ng/ml) for the 24 h and used for further analysis.

### Luciferase reporter assay

The amplified DNA sequences were cloned to the pmirGLO reporter plasmid (Promega, Madison, WI, USA) to form wildtype FUT2 3′UTR (WT) and mutated FUT2 3′-UTR (MUT) luciferase vectors. The pmiRGLO luciferase reporter vectors of the HOTAIR were constructed as above. Human chondrocytes were plated (5 × 10^4^ cells per well) in 24-well plates overnight and then were transfected with plasmid and miR-17-5p mimic or the control by using Lipofectamine 3000 (Invitrogen, Carlsbad, CA, USA). 48 h after transfection, the luciferase activity was measured by the dual-luciferase reporter gene assay system (Promega, Madison, WI, USA).

### RNA immunoprecipitation (RIP) assay

RIP assay was performed using the Magna RIPTM RNA Binding Protein Immunoprecipitation Kit (Millipore, Bedford, MA, USA) according to the manufacturer’s protocol. Briefly, cultured chondrocytes were collected and resuspended in RIP lysis buffer (Solarbio) then the cell extracts were incubated with RIP buffer containing magnetic beads conjugated with human anti-Ago2 antibody (Millipore) or mouse immunoglobulin G (IgG) control overnight at 4 °C. The next day, the magnetic beads were incubated with proteinase K after washing three times. Total RNAs was isolated from the extracts using the TRIzol reagent subsequently. Lastly, the relative enrichment of HOTAIR and miR-17-5p were determined by RT-qPCR analysis.

### Flow cytometry

The apoptotic rate of treated chondrocytes was determined by the PE Annexin V Apoptosis Detection Kit I (BD Pharmingen; San Jose, CA, USA) and then analyzed with a using a fluorescence-activated cell sorting (FACS) flow cytometer (BD Biosciences, San Diego, CA, USA). All experiments are repeated three times.

### CCK-8 assay

The proliferation capability of chondrocytes was assessed by Cell Counting Kit-8 (CCK-8, Sigma-Aldrich). In brief, 48 h after transfection, 10 μL of CCK-8 solution was added to each well and incubated for 4 h. Subsequently, the absorbance at 450 nm was measured using a microplate reader (Model 680; Bio-Rad, Hercules, CA, USA).

### Immunofluorescence staining

Chondrocytes were cultured in a 12-well plate and stimulated with IL-1β for 24 h. First, cells were fixed with 4% paraformaldehyde for 20 min. Subsequently, cells were permeabilized with 0.3% Triton X-100 for 10 min and then blocked with 5% BSA for 1 h. Then chondrocytes were incubated with primary antibody (Abcam, Cambridge, UK; Rabbit polyclonal antibody) overnight at 4 °C. The chondrocytes were washed with PBS and incubated with Alexa Fluor 488- or 594-conjugated goat anti-rabbit (Invitrogen) for 1 h at room temperature. Lastly, DAPI (Solarbio) was used for nuclear staining. Images were obtained on a fluorescence microscope.

### Immunohistochemical analysis

Human cartilage tissues and rats hind knee joints were fixed in 4% paraformaldehyde for paraffin embedding and cut into 4 μm slices. For immunohistochemical (IHC) staining, the sections were incubated with appropriate primary antibodies overnight at 4 °C after dewaxed in xylene. The next day, the slices were incubated with secondary streptavidin-horseradish peroxidase-conjugated antibody at room temperature for 60 min. Finally, the IHC signals were visualized using 3, 3′-diaminobenzidine (DAB) (ZLI9018, ZSGBBIO, China).

### Western blot

Cells were lysed on ice with RIPA lysis (Beyotime) buffer for 15 min and then protein concentration were measured by the BCA Assay Kit (Thermo Scientific). Protein fractions were subjected to 10% SDS-PAGE and then transferred onto polyvinylidene difluoride (PVDF) membrane (Millipore, Bedford, MA, USA). After blocked with 5% BSA for 2 h at room temperature, membranes were incubated with specific primary antibodies (Abcam, Cambridge, UK) overnight at 4 °C. The membranes were washed thrice and then incubated with HRP-conjugated secondary anti-rabbit antibodies for 1 h. All bands were detected using ECL Western blot kit (Beyotime, China).

### TUNEL assay

After 48 h of transfection and IL-1β stimulation, cells were fixed with 4% paraformaldehyde and permeabilized with equilibration buffer and 0.2% Triton X-100. DNA fragmentation was tested by TdT-mediated dUTP nick end labeling (TUNEL) as described by the manufacturer (promega, USA). Images were obtained on a fluorescence microscope.

### Rat model of OA

Animal experiments were performed according to the Guidelines for Animal Experimentation of Dalian Medical University and the rats were purchased from the Experimental Animal Center of Dalian Medical University. Experimental OA in 10-week-old SD rats was induced by medial collateral ligament transection and DMM as described previously. MiR-NC, miR-17-5p agomir (50 μM) and lentivirus (1 × 10^9^ PFU, 20 μl) expressing FUT2 or HOTAIR were injected into the knee joints of recipient rats 1 week after the surgery (20 μL per joint per rat two times a week for 4 weeks) (*n* = 6 per group). Eight weeks after surgery, rats were killed and knee joints were harvested.

### Statistical analysis

The data were presented as the mean ± SD as indicated. Statistical analysis was carried out using SPSS 20.0 (SPSS, Inc., Chicago, IL, USA). Statistical differences between two groups were determined by two-tailed Student’s *t*-test. *P* < 0.05 was considered statistically significant. *P* < 0.05 was considered statistically significant.

## Results

### Differential expression of HOTAIR, miR-17-5p and FUT2 in OA cartilage tissues

**Fig. 1:**
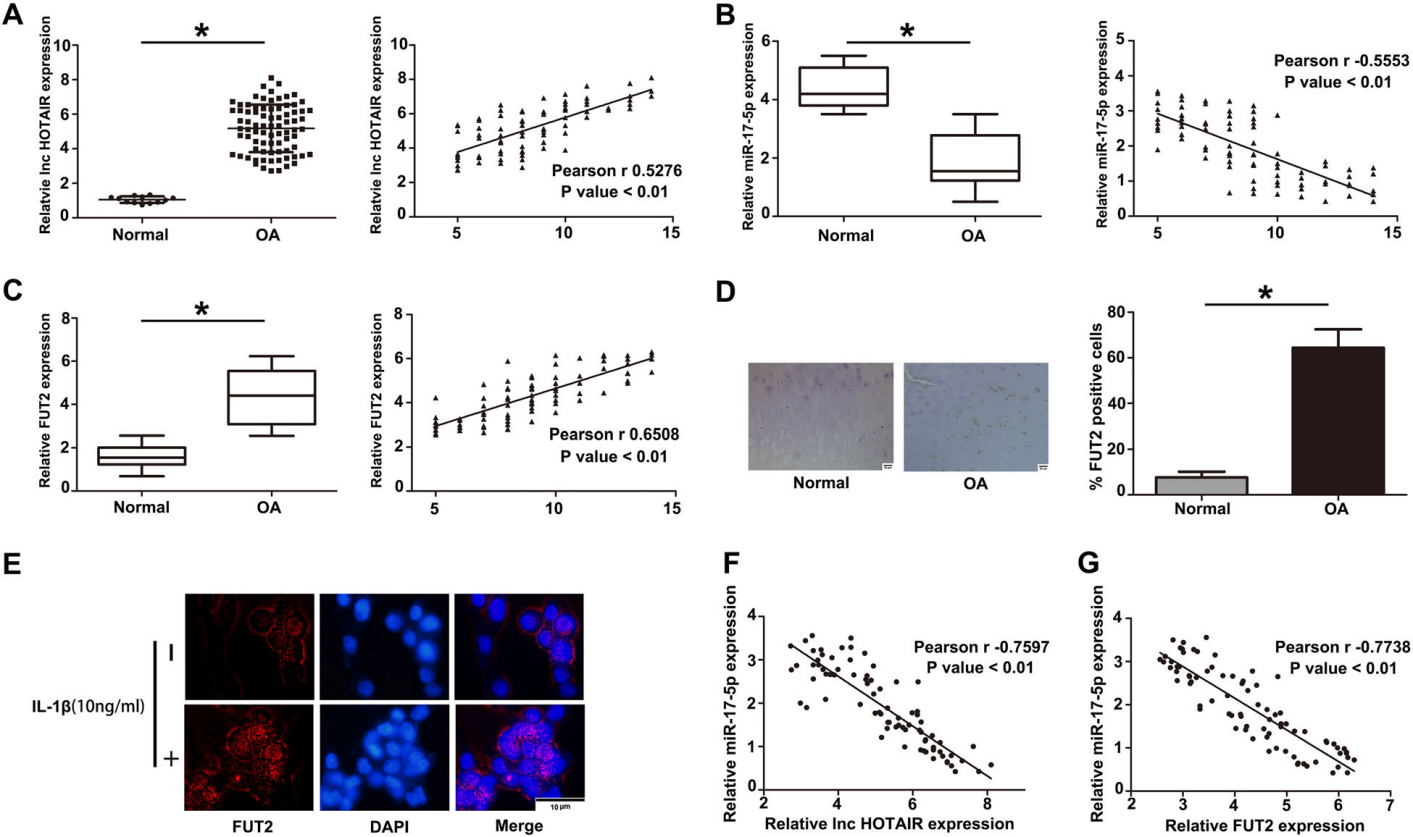
The differential expression of HOTAIR, miR-17-5p and FUT2 in clinical samples and human chondrocytes. **a** The expression of HOTAIR in healthy and OA human cartilage tissues was identified by RT-qPCR and was positively correlated with Mankin scale. **b** The level of miR-17-5p was lower in OA cartilage tissues compared with healthy cartilage tissues by RT-qPCR and were negatively correlated with Mankin scale. **c** FUT2 expression was determined in OA cartilage tissues and normal cartilage tissues by RT-qPCR. The level of FUT2 was positively correlated with Mankin scale. **d** Comparison on the tissues by IHC, the higher level of FUT2 was observed in OA tissues. **e** FUT2 expression was detected by immunofluorescence in chondrocytes incubated in the presence or absence of IL-1β at 10 ng/ml for 24 h. **f** The inverse relationship was observed between HOTAIR and miR-17-5p in OA cartilage tissues. **g** The miR-17-5p level was negatively correlated with FUT2 expression in OA cartilage. The data were means ± S.D. of three independent assays (*P < 0.05)

To investigate the expression of HOTAIR in cartilage tissues, qRT-PCR was performed in samples from 80 OA patients and 12 healthy subscribers. HOTAIR expression was significantly higher in OA cartilage tissues than that in normal tissues. In addition, to evaluate OA severity, modified Mankin grading was performed. In the modified Mankin grading, abnormalities in structure (0–6 points), cellularity (0–3 points) and Safranin-O staining (0–4 points) were assessed up to a maximum score of 13 points. The expression of HOTAIR was positive correlated with modified Mankin scores (Fig. 1a). MiR-17-5p level was decreased in OA cartilage and had a negatively correlation with modified Mankin scores (Fig. 1b). The mRNA and protein levels of FUT2 were up-regulated in OA cartilage tissues and IL-1β induced chondrocytes (Fig. 1c–e). Furthermore, the expression of miR-17-5p was negatively correlated with HOTAIR and FUT2 (Fig. 1f, g). These results indicated that HOTAIR/ miR-17-5p/ FUT2 might play an important role in OA progression.

### HOTAIR promotes IL-1β-induced ECM degradation, cell apoptosis and inhibits cell proliferation in OA progression

**Fig. 2:**
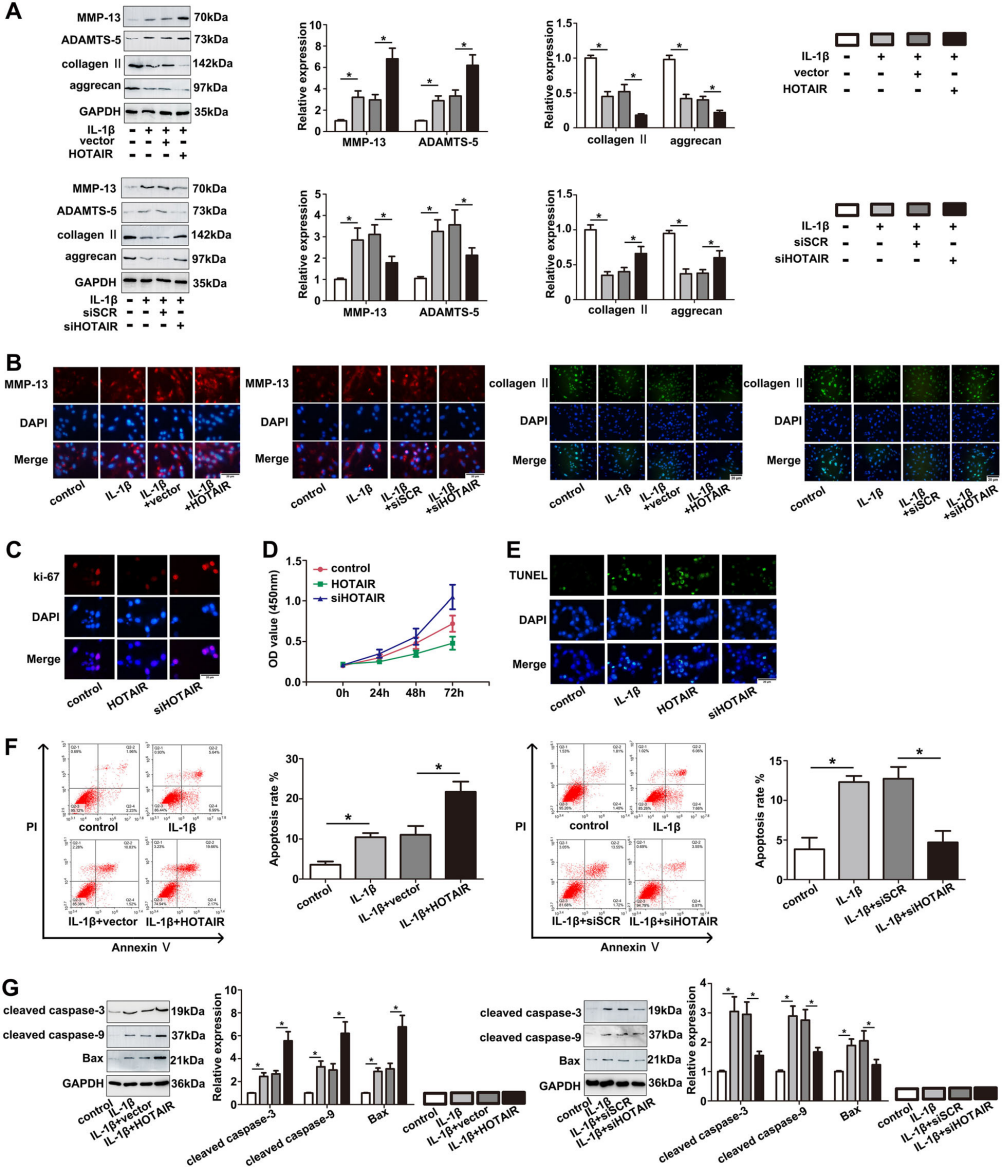
Functional investigation of HOTAIR in chondrocytes. **a** Representative catabolic and anabolic proteins, MMP-13, ADAMTS-5, type II collagen and aggrecan were analyzed by western blot in human chondrocytes incubated in the presence or absence of IL-1β at 10 ng/ml for 24 h after transfection with HOTAIR or siHOTAIR. **b** MMP-13 and type II collagen levels in chondrocytes transfected with HOTAIR or siHOTAIR were analyzed by immunofluorescence staining. **c**, **d** Ki67 immunofluorescence staining and CCK-8 proliferation assay were used to identify the proliferative capability of chondrocytes after transfecting with HOTAIR or siHOTAIR. **e**, **f** 24 h after trasnfection and IL-1β stimulation, TUNEL assays and flow cytometry were performed to evaluate apoptosis of chondrocytes. **g** 24 h after trasnfection and IL-1β stimulation, Bax, cleaved caspase-3 and cleaved caspase-9 levels were measured by western blot. Each bar represented mean ± SD (*n* = 3). *P < 0.05 vs. control

To explore the role of HOTAIR on IL-1β-induced ECM degradation, chondrocytes were treated with IL-1β (10 ng/ml) for 24 h after transfection. The protein levels of major cartilage-related genes were tested, including MMP-13, ADAMTS-5, type II collagen and aggrecan. As shown in Fig. 2a, the protein levels of typical cartilage-degrading enzymes (MMP-13 and ADAMTS-5), were markedly increased after HOTAIR overexpression. On the contrary, the expression of type II collagen and aggrecan, two kinds of main cartilaginous extracellular matrix proteins contributing to cartilage formation, were significantly reduced in overexpressed HOTAIR group compared with IL-1β group. These findings were further validated by immunofluorescence staining (Fig. 2b). In addition, the role of HOTAIR on the biologic activity of chondrocytes was also explored. As shown in Fig. 2c, d, the result of ki-67 immunofluorescence staining and CCK-8 assay demonstrated that the proliferative ability of chondrocytes was attenuated after HOTAIR overexpression and supression of HOTAIR could promote cell proliferation. Furthermore, chondrocytes apoptosis was detected by TUNEL assay, flow cytometry and western blot (Fig. 2e–g). After transfection with HOTAIR, a higher proportion of Annexin V-positive cells were shown and the protein levels of cleaved caspase-3, cleaved caspase-9 and bax were up-regulated. On the contrary, knockdown of HOTAIR led to a lower proportion of Annexin V-positive cells. Consistent with the result of flow cytometry, the decreased levels of cleaved caspase-3, cleaved caspase-9 and bax were detected by western blot after transfecting with siHOTAIR. Taken together, we believed that HOTAIR could mediate OA progression by regulating ECM degradation, chondrocytes proliferation and apoptosis.

### HOTAIR acts as a ceRNA by sponging miR-17-5p and indirectly regulates FUT2 expression

**Fig. 3:**
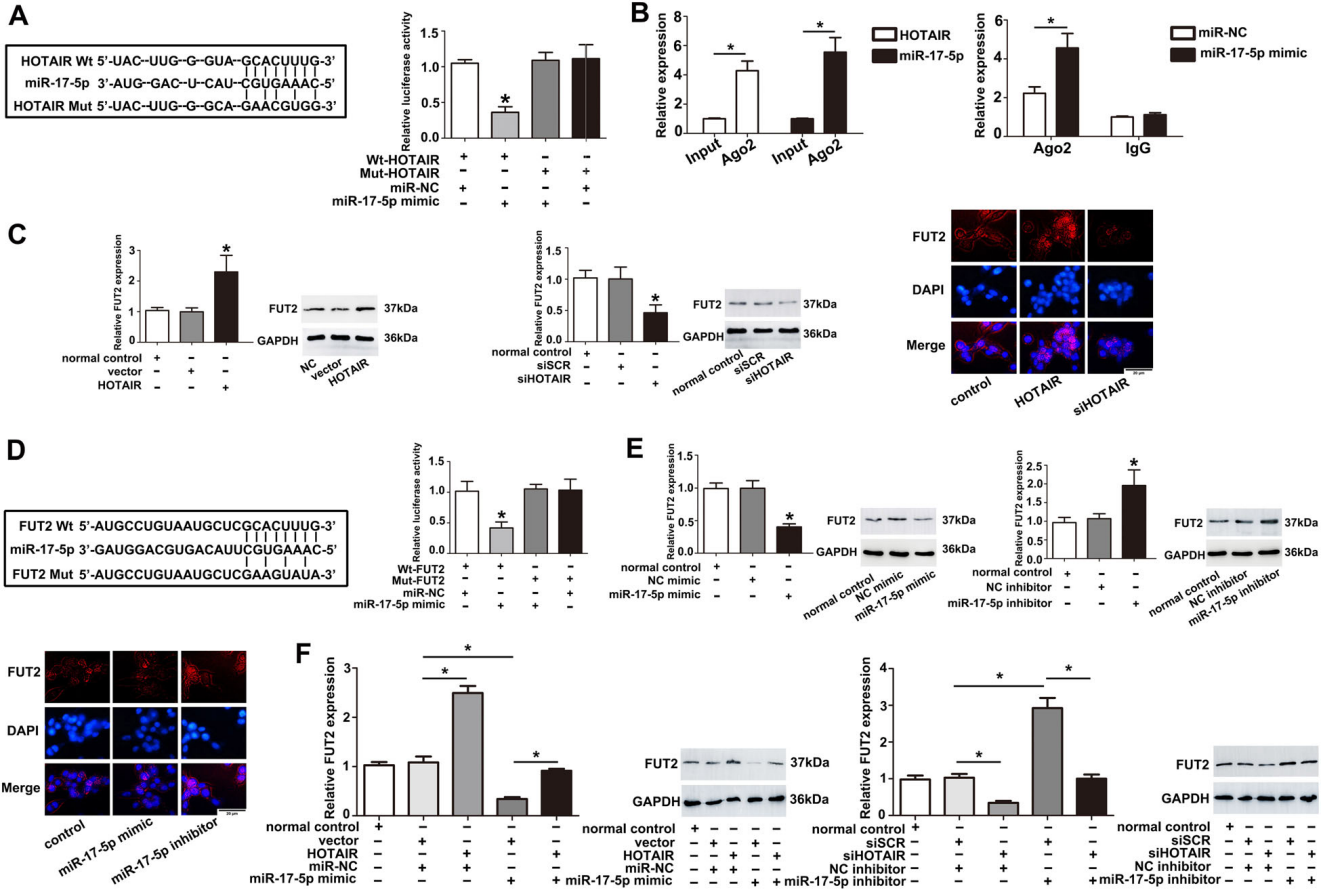
HOTAIR acted as a ceRNA by sponging miR-17-5p and regulated FUT2 expression indirectly. **a** The predicted binding sites of miR-17-5p to HOTAIR sequence and the luciferase activity of chondrocytes co-transfected with miR-17-5p mimic and luciferase reporters containing HOTAIR-Wt or HOTAIR-Mut transcript were shown. **b** RNA immunoprecipitation was performed in chondrocytes transfected with miR-NC and miR-17-5p mimic. HOTAIR expression was detected by using qRT-PCR. RNA levels were presented as fold enrichment in Ago2 relative to IgG immunoprecipitates. **c** FUT2 protein was detected by western blot and immunofluorescence after transfected with HOTAIR or siHOTAIR. **d** The predicted binding sites of miR-17-5p to HOTAIR sequence and the luciferase activity of chondrocytes co-transfected with miR-17-5p mimic and luciferase reporters containing FUT2-Wt or FUT2-Mut transcript were shown. **e** FUT2 protein was detected by western blot and immunofluorescence staining after transfecting with miR-17-5p mimic or miR-17-5p inhibitor. **f** FUT2 protein was detected by western blot after co-transfecting with HOTAIR and miR-17-5p mimic or siHOTAIR and miR-17-5p inhibitor. The data were means ± S.D. of three independent assays (*P < 0.05)

Several studies reported that HOTAIR could bind directly to miRNA and function as sponges or ceRNAs to control the availability of miRNA for binding to their target mRNAs. To further investigate the underlying mechanism of HOTAIR in OA, we used online software miRcode to research for the miRNAs interacted with HOTAIR. Interestingly, we predicted the potential miRNA binding sites in HOTAIR and found that miR-17-5p was among the numerous possible targets of HOTAIR. The relationship of HOTAIR and miR-17-5p was further validated by dual luciferase reporter assays, which verified the surmise that HOTAIR was directly targeted by miR-17-5p in chondrocytes (Fig. 3a). Argonaute2 (Ago2) protein is a key components of the RNA induced silencing complex (RISC) and Ago2 antibody is useful in capturing mature miRNAs^23^. Therefore, anti-Ago2 RIP assay was used to elucidate the potentially endogenous interaction between HOTAIR and miR-17-5p. The results showed that HOTAIR and miR-17-5p were preferentially enriched in the Ago2 pellet relative to control IgG immunoprecipitates and endogenous HOTAIR pull-down was specifically enriched in miR-17-5p-transfected cells (Fig. 3b). The mRNA and protein levels of FUT2 were markedly increased after HOTAIR overexpression (Fig. 3c). The bioinformatics analyses (TargetScan, miroRNA.org and Starbase v2.0) were performed to search for miR-17-5p potential target mRNAs and initially identified FUT2 as a potential target. The dual luciferase reporter assays demonstrated FUT2 was a direct target of miR-17-5p (Fig. 3d). MiR-17-5p mimic significantly suppressed the expression of FUT2 in chondrocytes (Fig. 3e). Moreover, FUT2 expression was significantly decreased after transfecting with siHOTAIR, and the inhibitory effect of siHOTAIR was notably reversed by co-transfection with miR-17-5p inhibitor in chondrocytes (Fig. 3f). On the contrary, HOTAIR overexpression increased FUT2 level, and this effect could be reversed by co-transfection with miR-17-5p mimic. These results suggested that HOTAIR functioned as a ceRNA by sponging miR-17-5p and indirectly regulated FUT2 expression.

### MiR-17-5p reverses HOTAIR-mediated ECM degradation, anti-proliferation and pro-apoptosis in IL-1β-induced chondrocytes

**Fig. 4:**
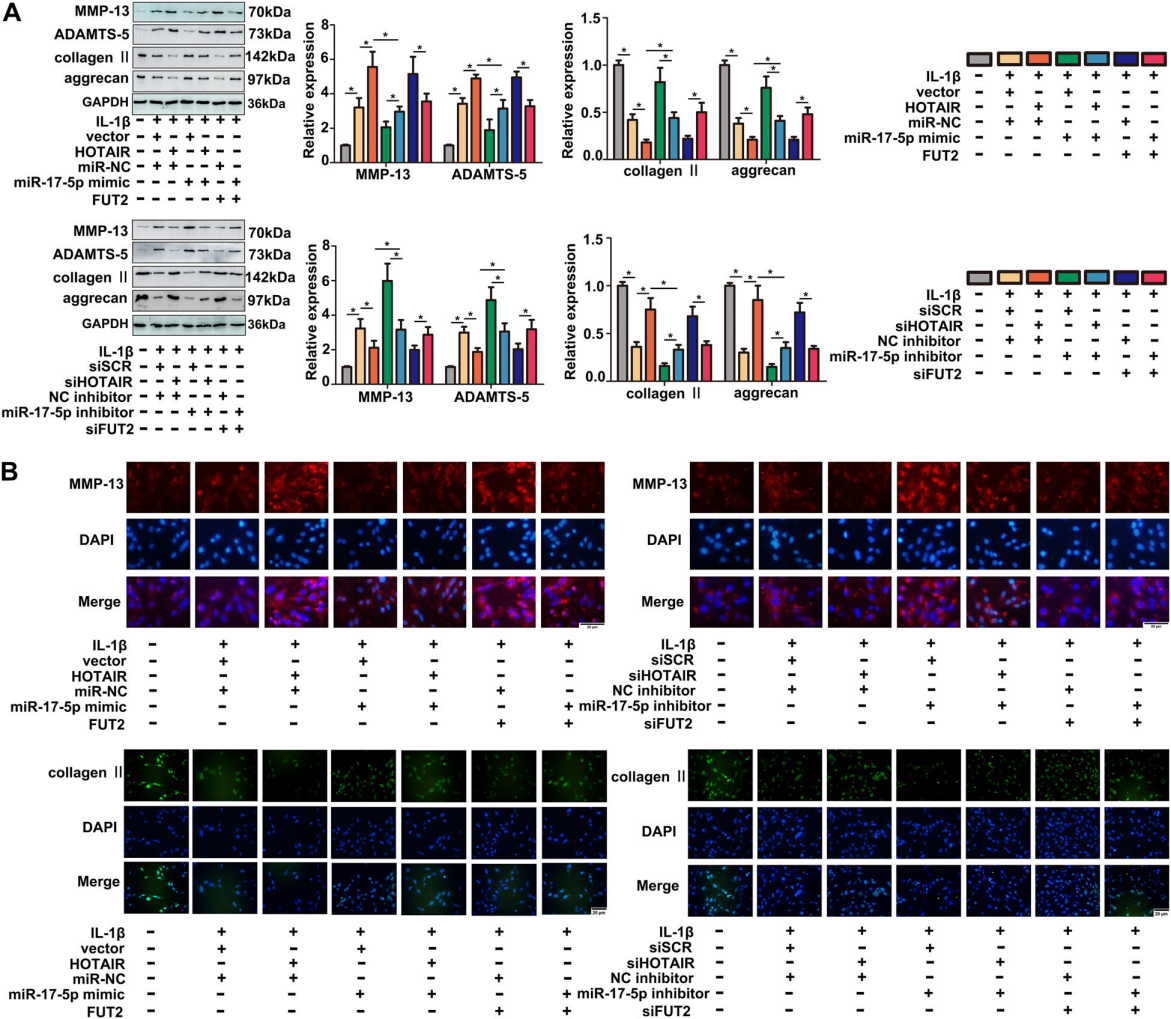
MiR-17-5p reversed HOTAIR-mediated ECM degradation. **a** MMP-13, ADAMTS-5, type II collagen and aggrecan levels were detected by western blot after transfection and IL-1β stimulation. **b** MMP-13 and type II collagen levels were detected by immunofluorescence staining after transfection and IL-1β stimulation. Each bar represented mean ± SD (*n* = 3). *P < 0.05 vs. control

**Fig. 5:**
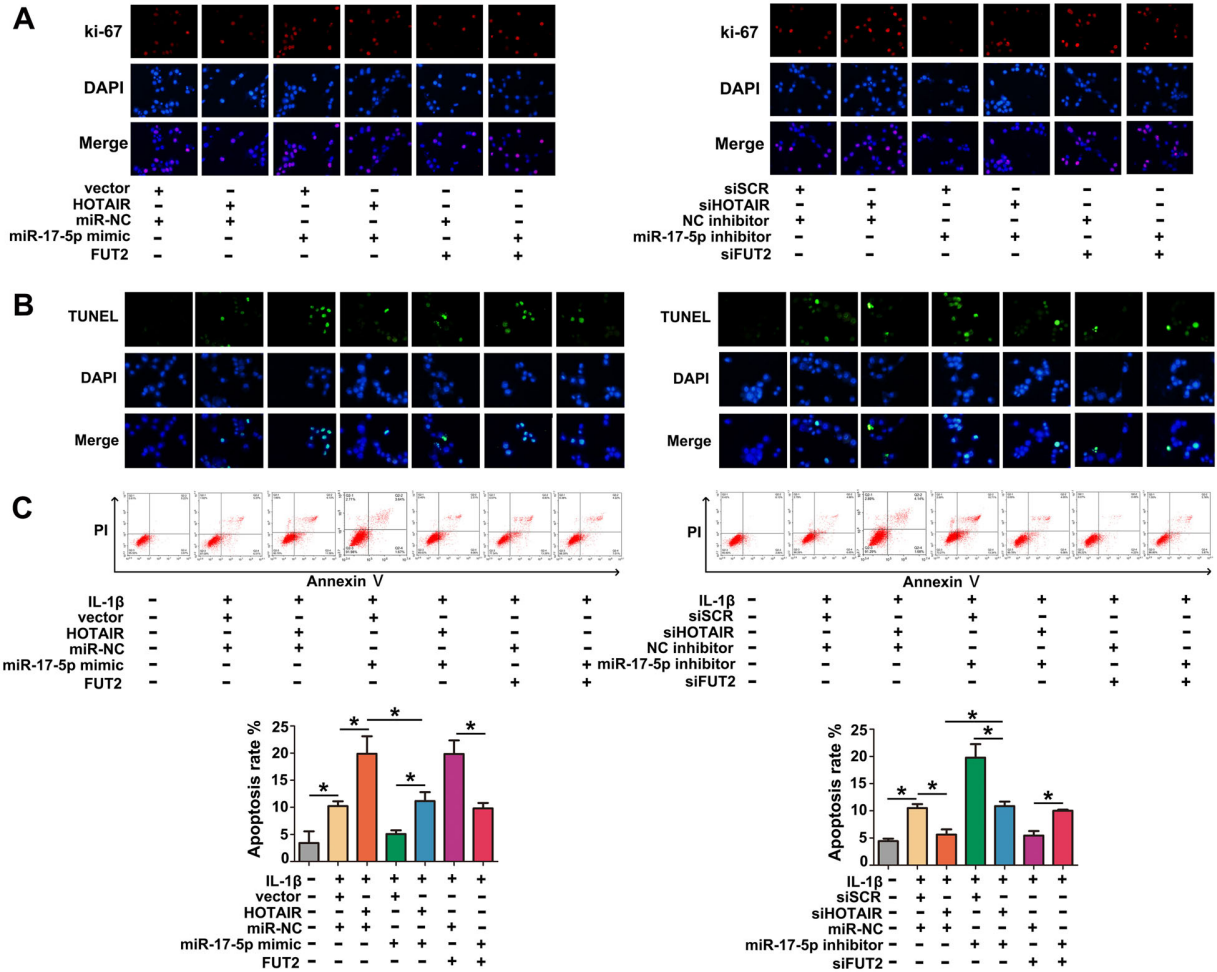
MiR-17-5p reversed HOTAIR-mediated anti-proliferation and pro-apoptosis in IL-1β-induced chondrocytes. **a** The proliferative ability of chondrocytes was measured by ki67 immunofluorescence staining. **b**, **c** Chondrocytes apoptosis was detected by TUNEL assay and Flow cytometry. Each bar represented mean ± SD (*n* = 3). *P < 0.05 vs. control

**Fig. 6:**
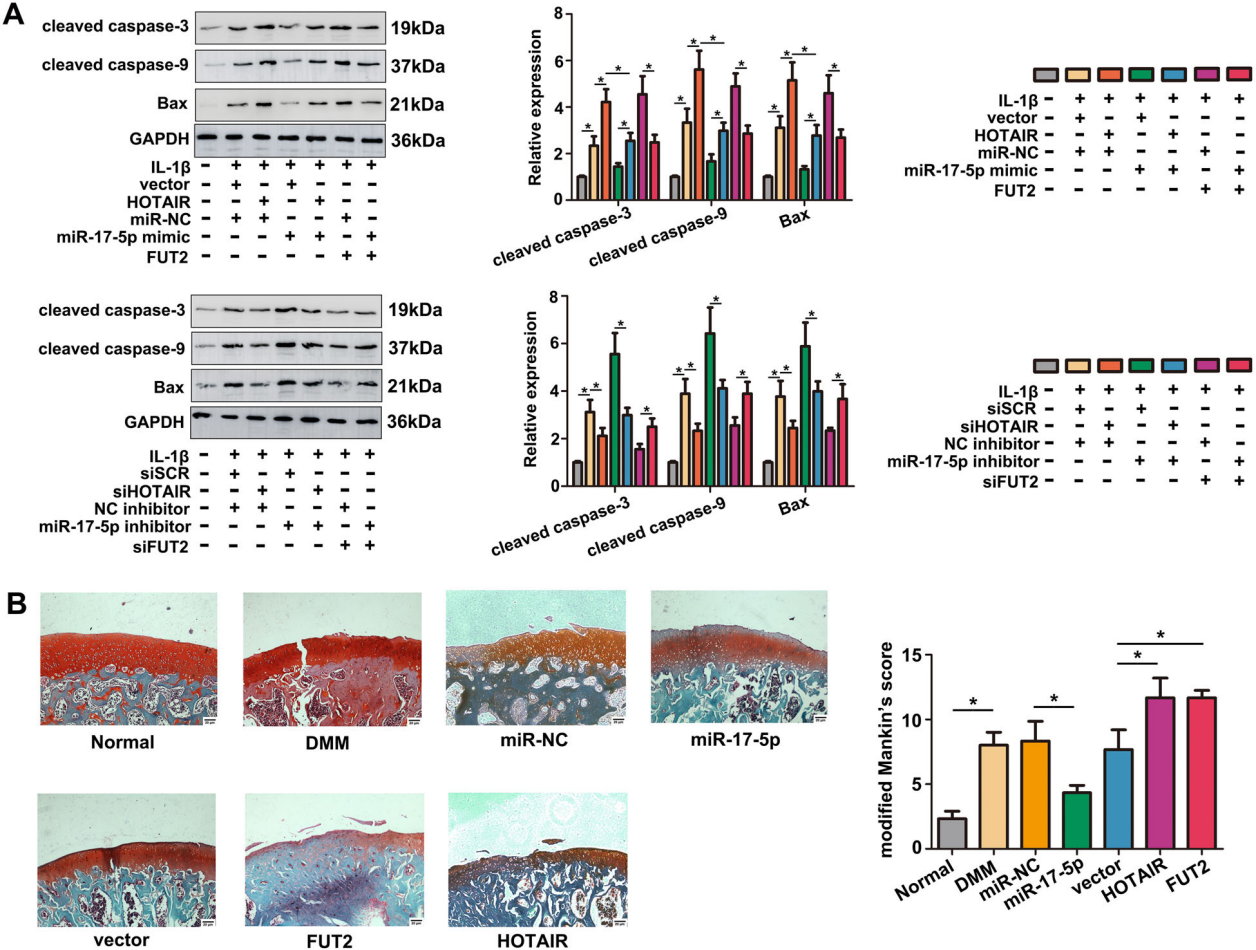
The effects of HOTAIR/miR-17-5p/FUT2 on apoptotic molecules and cartilage degradation in vivo. **a** Cleaved caspase-3, cleaved caspase-9 and Bax levels were detected by western blot after tansfection and IL-1β stimulation. **b** Histologic section of cartilage structure was stained by safranin-O in each group. The histologic scores were assessed using modified mankin’s scale. Each bar represented mean ± SD (*n* = 3). *P < 0.05 vs. control

To explore whether HOTAIR exerted its function by miR-17-5p in chondrocytes the rescue experiments were performed. As shown in Fig. 4a, b, up-regulation of HOTAIR or FUT2 significantly promoted ECM degradation, while the effect could be inhibited by miR-17-5p overexpression. Moreover, miR-17-5p overexpression dramatically inhibited ECM degradation induced by IL-1β. On the contrary, knockdown of HOTAIR or FUT2 decreased the levels of MMP-13 and ADAMTS-5 proteins, while suppression of miR-17-5p blocked the effect effectively. These results indicated that HOTAIR contributed to ECM degradation by suppressing miR-17-5p or increasing FUT2. Interestingly, HOTAIR/FUT2 mediated pro-apoptosis and anti-proliferation effect was evidently reversed by miR-17-5p mimic. In contrast, si-HOTAIR/siFUT2 mediated anti-apoptosis effect was significantly reversed by miR-17-5p knockdown, and si-HOTAIR/siFUT2 mediated pro-proliferation was strikingly abrogated by miR-17-5p inhibitor (Fig. 5a–c). Moreover, western blot analysis also verified the expressional changes of cleaved caspase-3, cleaved caspase-9 and Bax (Fig. 6a). We also detected the effect of HOTAIR/miR-17-5p/FUT2 on cartilage in vivo. The rat intra-articular injection with miR-17-5p agomir showed a better performance on superficial layer with decreased cartilage surface erosion and increased safranin-O staining in the superficial layer, as compared with miR-NC group. In contrary, the result of HOTAIR or FUT2 overexpression group showed a severe damage on superficial layer compared with vector group (Fig. 6b). These results suggested that HOTAIR might exert its function by sponging miR-17-5p and indirectly regulating FUT2 in OA.

### FUT2 aggravates OA progression through wnt/β-catenin pathway

**Fig. 7:**
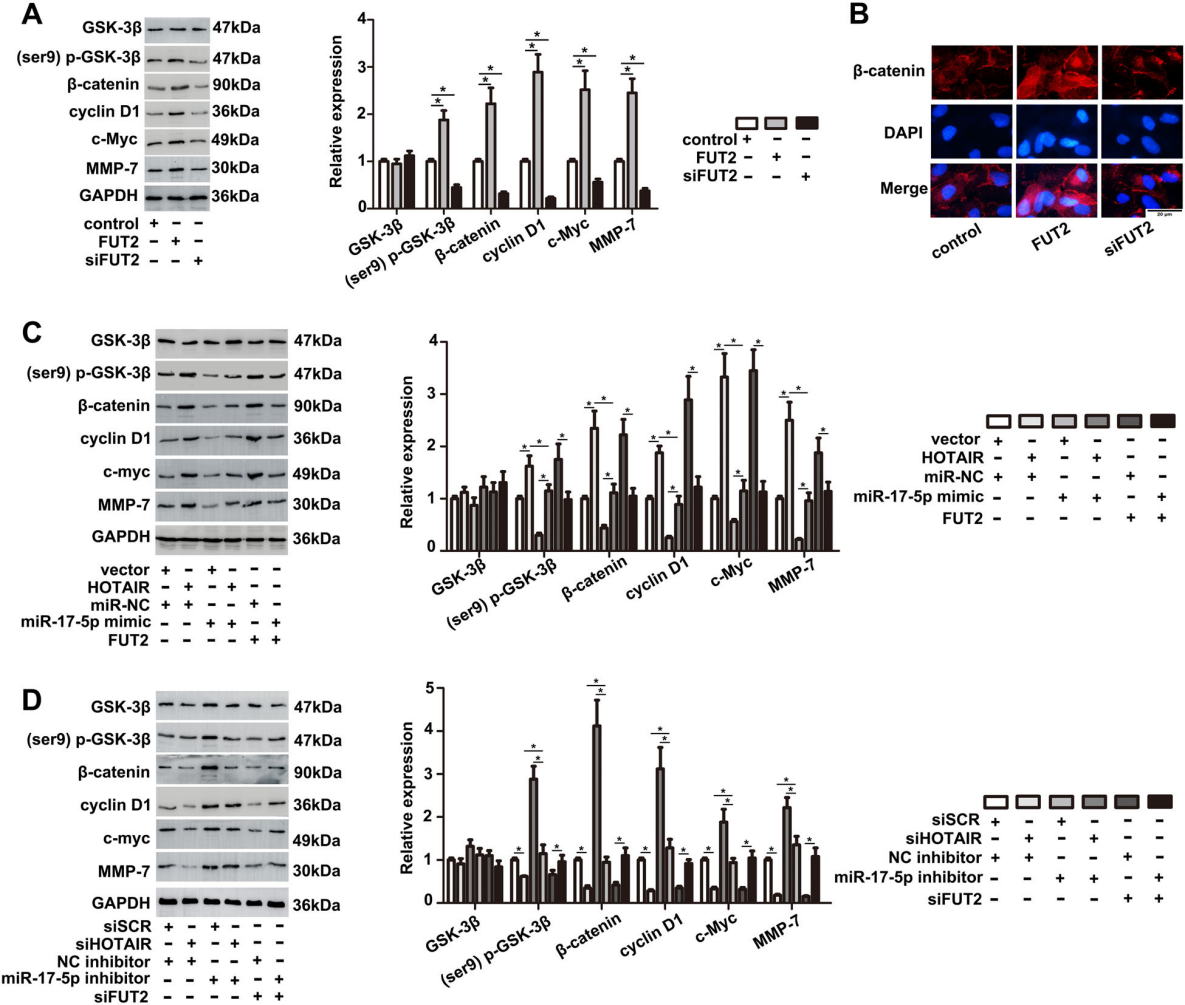
The effect of HOTAIR/miR-17-5p/FUT2 axis on wnt/β-catenin pathway. **a**, **b** The effect of FUT2 on wnt/β-catenin pathway was detected by western blot and immunofluorescence staining. The data are means ± S.D. of three independent assays (*P < 0.05). **c**, **d** The effect of HOTAIR/miR-17-5p/FUT2 axis on wnt/β-catenin pathway was detected by western blot. The data were means ± S.D. of three independent assays (*P < 0.05)

Many literatures have reported FUT family activated wnt/β-catenin pathway in a variety of biological processes^24^. In order to figure out the function of molecular mechanisms induced by HOTAIR, miR-17-5p and FUT2, we wondered FUT2 could exert its posttranslational modification function and participate in wnt/β-catenin pathway activation process. As shown in Fig. 7a, the overexpression of FUT2 increased the protein levels of p-GSK-3β and total β-catenin in chondrocytes and promoted the expression of downstream molecules, such as c-myc, cyclin D1 and MMP-7. To further detect nuclear and cytoplasmic β-catenin protein levels in chondrocytes upon FUT2 overexpression, immunofluorescence assay was performed (Fig. 7b). The result demonstrated FUT2 regulated the nuclear β-catenin expression and increased β-catenin nuclear accumulation in chondrocytes. As shown Fig. 7c–d, higher expression of p-GSK-3β and total β-catenin was observed in overexpressed HOTAIR or FUT2 group, and miR-17-5p could reverse this effect. On the contrary, the degree of wnt/β-catenin pathway was decreased in cells transfected with siHOTAIR or siFUT2. These data suggested that the HOTAIR/miR-17-5p/FUT2 mediated OA progression via wnt/β-catenin pathway.

## Discussion

OA is becoming a major public health problem and the underlying mechanism is not fully learned. Recently, more and more researchers noticed that lncRNAs play important roles in OA progression. Several lncRNAs were identified to contribute to OA progression, including GAS5, PCGEM1, lncRNA-CIR and HOTAIR^25–27^. LncRNA HOTAIR was up-regulated and contributed to IL-1β-induced MMP overexpression, suggesting an important pro-apoptotic role of HOTAIR in OA chondrocytes^28^. HOTAIR strongly promoted the expression of ADAMTS-5 by increasing its mRNA stability in human OA articular chondrocytes, suggesting that HOTAIR was a new therapeutic target for ADAMTS-5 inhibition in human OA cartilage^29^. Although some studies have reported HOTAIR expression was up-regulated during OA progression, the underlying molecular mechanism has not been widely studied. In the present study, we found HOTAIR expression was markedly increased in OA cartilage tissues and has a positive correlation with the severity of OA. Moreover, functional investigation revealed HOTAIR contributed to IL-1β-induced ECM degradation and cell apoptosis and the up-regulated HOTAIR also inhibited cell proliferation. These data suggested that HOTAIR might perform an important function in OA.

CeRNA hypothesis has gained substantial attention as an alternative function for lncRNAs^30^. The novel regulatory mechanism has been identified in which crosstalk between lncRNAs and mRNAs occurred by competing for shared miRNAs response elements. LncRNA MEG3 promoted chondrocytes apoptosis and inhibited cell proliferation through miR-16/SMAD7 axis^31^. LncRNA RP11-445H22.4 acting as a ceRNA against miR-301a, aggravated IL-1β-induced injury in OA progression^32^. In regard to HOTAIR, lots of studies verified that HOTAIR exerted its function by antagonizing the miRNAs effects and regulating the expression of miRNAs endogenous targets in many diseases. HOTAIR/miR-20a-5p/HMGA2 axis influenced cell growth, migration, invasion and apoptosis in breast cancer^33^. HOTAIR also regulated NPM1 via interacting with miR-646, thereby governing the viability, migration and invasion of endometrial cancer^34^. Our current research observed an inverse correlation between HOTAIR and miR-17-5p, which stimulated our interest to determine whether there was a ceRNA mechanism involved between HOTAIR and miR-17-5p. We found that the expression of HOTAIR and FUT2 was inversely correlated to miR-17-5p in OA cartilage tissues. Altered HOTAIR and miR-17-5p could regulate FUT2 level. HOTAIR acted as a ceRNA by sponging miR-17-5p and indirectly regulated FUT2 expression. Furthermore, HOTAIR promoted ECM degradation and cell apoptosis by indirectly up-regulating FUT2 expression, and this effect could be reversed by miR-17-5p. To the best of our knowledge, we firstly demonstrated HOTAIR/miR-17-5p/FUT2 axis mediated OA progression.

Glycosylation is one of the common post-translational modification forms and is involved in a variety of physiological and pathological processes^35^. Fucosylation is a kind of glycosylation, which catalyzed by FUTs. Recently, some researchers reported that aberrant fucosylation and FUTs played an important role in the activation of wnt/β-catenin pathway in various diseases. Yang et al. suggested that FUT8 promoted breast cancer cell stemness and epithelial-mesenchymal transition by activating wnt/β-catenin pathway^36^. Zhang et al.^37^ demonstrated FUT4 promoted embryo adhesion and implantation via wnt/β-catenin signaling pathway. In this study, we found that FUT2 was up-regulated during OA progression and validated FUT2 could increase β-catenin nuclear accumulation and activate wnt/β-catenin pathway. Several studies also reported HOTAIR activated wnt/β-catenin pathway via multiple ways. Cheng et al. reported knockdown of HOTAIR inhibited wnt/β-catenin pathway by up-regulating miR-34a in gastric cancer cells^38^. Li et al.^39^ elucidated overexpression of HOTAIR contributed to chemoresistance by wnt/β-catenin pathway in ovarian cancer. Our data demonstrated HOTAIR increased the activity of wnt/β-catenin pathway by sponging miR-17-5p to indirectly regulate FUT2 in chondrocytes, suggesting a new sight in OA progression.

In conclusion, HOTAIR was up-regulated in OA cartilage tissues and the over-expressed HOTAIR aggravated chondrocytes injury and apoptosis, suggesting it could act as a useful marker and potential therapeutic target in OA. We further identified HOTAIR exerted its effects partially through the HOTAIR/miR-17-5p/FUT2/β-catenin axis. This finding improved understanding of mechanism involved in OA progression and provided novel targets for the molecular treatment for OA.
